# Mechanistic study of *TFE3* breakage in *TFE3*-rearranged renal cell carcinoma: the perspective of non-canonical DNA structures and their stability

**DOI:** 10.3389/fgene.2025.1694739

**Published:** 2025-11-25

**Authors:** Xiaopo Zhang, Qinshuang Dang, Xinghe Pan, Zhenggen Deng, Yanhao Xu, Weidong Gan, Hongqian Guo

**Affiliations:** 1 Department of Urology, Nanjing Drum Tower Hospital, Nanjing Drum Tower Hospital Clinical College, Nanjing Medical University, Nanjing, China; 2 Department of Urology, Nanjing Drum Tower Hospital, Nanjing Drum Tower Hospital Clinical College, Nanjing University of Chinese Medicine, Nanjing, China; 3 Department of Urology, Nanjing Drum Tower Hospital, Affiliated Hospital of Medical School, Nanjing University, Nanjing, China

**Keywords:** TFE3-rearranged renal cell carcinoma, chromosome breakpoint, non-canonical DNA structure, gibbs free energy, genome instability

## Abstract

**Background/Objectives:**

To address the unelucidated mechanisms of breakpoint formation in *TFE3*-rearranged renal cell carcinoma (*TFE3*-rRCC), this study characterizes breakpoint distribution within the *TFE3* gene. We further explore how non-canonical DNA structures and their thermodynamic stability fluctuation may act as predisposing factors for the genomic instability driving these characteristic translocations.

**Methods:**

*TFE3* breakpoints were identified in a cohort of 31 *TFE3*-rRCC tumor samples. The chi-square test was used to assess the statistical significance of breakpoint clustering. To investigate potential structural determinants, we predicted the distribution of G-quadruplex-forming sequences and palindromic motifs. Moving beyond simple motif density, we calculated the local Gibbs free energy changes (*ΔG*) associated with DNA secondary structures using *Mfold* and *RNAfold* to model thermodynamic stability across the *TFE3* gene. This thermodynamic stability fluctuation was quantified as the maximum absolute local change in folding free energy (|d*ΔG*|). Finally, this correlation between thermodynamic stability fluctuation and breakpoint location was validated by analyzing the 13 most frequently rearranged genes reported in the COSMIC database.

**Results:**

A significant breakpoint cluster was identified within intron 5 of *TFE3*, containing 23 of 31 breakpoints (74.19%; chi-square test, *P* < 0.05). While the simple density of G-quadruplex or palindromic motifs did not directly correlate with breakpoint locations, a strong association with local thermodynamic stability fluctuation was observed. The region within intron 5 exhibited the highest thermodynamic stability fluctuation. This result suggests that regions of high thermodynamic stability fluctuation are correlated with increased susceptibility to DNA breakage. This finding was corroborated in the COSMIC dataset, where breakpoints in 12 of the 13 most frequently rearranged genes were similarly located near peaks of high |d*ΔG*|.

**Conclusion:**

Our findings indicate that breakpoint events in *TFE3*-rRCC are non-randomly clustered within intron 5. This clustering correlates strongly with regions characterized by high thermodynamic stability fluctuation (|d*ΔG*|) of potential non-canonical DNA secondary structures. The principle that elevated local thermodynamic stability fluctuation is a feature of breakpoint locations was supported by analysis of a broader set of oncogenes, suggesting that high local thermodynamic stability fluctuation is a common feature of translocation-prone regions in cancer, representing a plausible, though not proven, contributor to genomic fragility.

## 1 Introduction

*Transcription Factor Binding to IGHM Enhancer 3*-rearranged renal cell carcinoma (*TFE3*-rRCC) is a distinct subtype of renal cell carcinoma that predominantly affects young and middle-aged patients and is characterized by chromosome translocations involving the *Transcription Factor E3* (*TFE3*) gene (HGNC:11752) (Pinto and Chetty, 2020). These tumors exhibit a variety of *TFE3* gene fusions, with partners including *ASPSCR1, CLTC, DVL2, LUC7L3, KHSRP, PRCC, PARP14, NONO, SFPQ1, MED15, RBM10, NEAT1*, and *KAT6A* (Pei et al., 2019). The resulting chimeric *TFE3* proteins, generated by these chromosomal rearrangements, retain the bHLH-Zip domain and act as oncogenic transcription factors (Tang and Baba, 2023). Consequently, disruption of the *TFE3* gene is considered to be the initiating oncogenic event.

The *TFE3* gene is located within FRAXG, a common fragile site (CFS) (Mrasek et al., 2010). CFSs are specific, heritable chromosomal regions that exhibit a high susceptibility to forming gaps or breaks when cells are subjected to replicative stress, such as exposure to DNA replication inhibitors (Mirceta et al., 2022). The fragility of these sites is a major contributor to genomic instability. Over half of the breakpoints in gene pairs involved in cancer-specific recurrent translocations have been mapped to fragile human chromosomal sites. Among the 25 genes involved in *TFE3*-rRCC (*TFE3* and its fusion partners), 16 (64%) are located within known human chromosomal fragile sites (Supplementary File 1). While multiple mechanisms, such as R-loops and replication timing, contribute to genomic fragility, this study focuses on the role of thermodynamic stability fluctuation arising from non-canonical DNA structures (Sinai et al., 2019). Since then, more than 15 types of DNA structure that differ from canonical B-DNA have been reported, including hairpins/cruciform, Z-DNA, triplexes, tetraplexes, slipped DNA, and sticky DNA and so on (Wang and Vasquez, 2023).

AT-rich DNA sequences, known for their structural flexibility, can promote the formation of secondary structures that impede replication fork progression, leading to stalling and an increased propensity for DNA breakage at these loci (Sinai et al., 2019; Duardo et al., 2023). During DNA replication, transient single-stranded DNA (ssDNA) regions are exposed, particularly on the lagging-strand template. These exposed ssDNA regions are thermodynamically unstable and can fold into non-canonical secondary structures, such as hairpins (formed from palindromic sequences) and G-quadruplexes, to achieve a more stable, lower-energy conformation. This folding process releases energy that is quantifiable as Gibbs free energy change (*ΔG*). The thermodynamic stability of these structures can be quantified by the change in Gibbs free energy (*ΔG*) at physiological temperature (37 °C), providing insight into their potential for formation (Takahashi et al., 2022). *ΔG* = *ΔH* − T*ΔS*, where *ΔH* represents the change in enthalpy, T is the temperature, and *ΔS* is the change in entropy. A more negative *ΔG* value indicates a more spontaneous folding process and a more stable resulting structure.

Currently, investigations into the link between thermodynamic stability fluctuation and chromosomal translocations have been largely correlational and lack validation at the nucleotide-resolution of precise breakpoints. This study aims to address this gap by analyzing the thermodynamic landscape at the precise breakpoints of the *TFE3* gene in *TFE3*-rRCC patients, thereby elucidating a potential biophysical mechanism underlying *TFE3* gene disruption.

## 2 Materials and methods

### 2.1 Detecting *TFE3* breakpoints in *TFE3*-rRCC patients using second-generation sequencing technology

This retrospective study was approved by the Research Ethics Committee of Nanjing Drum Tower Hospital, and a waiver of informed consent was granted for all included cases. A total of 31 formalin-fixed, paraffin-embedded tumor tissue samples, with a diagnosis of *TFE3*-rearranged renal cell carcinoma (*TFE3*-rRCC) confirmed by *TFE3* immunohistochemistry and break-apart fluorescence *in situ* hybridization (FISH) probes, were selected from the case database of Nanjing Drum Tower Hospital (2018–2024). These 31 FFPE tumor tissue blocks were profiled using both Illumina RNA sequencing (RNA-seq) and DNA targeted gene capture sequencing to characterize the underlying *TFE3* fusion events. Subsequently, the sequencing data were analyzed to identify the specific fusion partner genes and to precisely map the genomic breakpoints.

**FIGURE 1:**
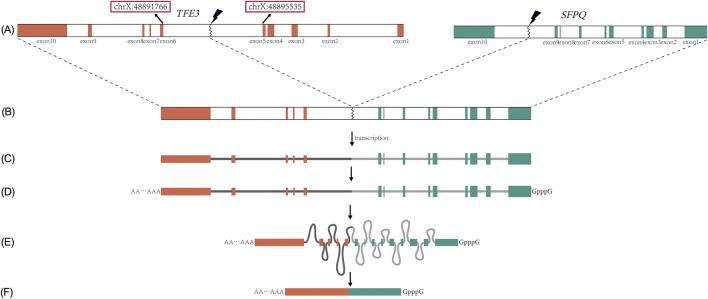
Fusion, transcription, and splicing of the *TFE3* and its partner gene (illustrated by SFPQ). **(A)** Breakage of the *TFE3* and SFPQ, with the red box indicating the 5th intron start/end; **(B)** SFPQ-*TFE3* fusion gene; **(C)** Transcriptional precursor, i.e., pre-mRNA; **(D)** Modification of the start and end of pre-mRNA; **(E)** Formation of lariat RNA during splicing, loss of breakage information at the intron; **(F)** mRNA appearing in the cytoplasm, with the lariat removed.

While RNA sequencing (RNA-seq) can identify the transcriptomic fusion junction by detecting the chimeric mRNA sequence, this junction does not precisely delineate the genomic breakpoint. Specifically, when a genomic breakpoint occurs within an intron, the process of pre-mRNA splicing removes the intron-containing sequence, thereby eliminating the breakpoint information from the mature mRNA transcript (Figure 1). Consequently, RNA-seq can reveal which exons are involved in the fusion but can only approximate the location of the genomic breakpoint to the flanking introns. Similarly, for breakpoints occurring within an exon, RNA-seq can detect the fusion site, but it cannot resolve the exact genomic coordinates or account for small insertions and deletions (indels) that may arise during the DNA repair process.

Bioinformatic analysis commenced with quality control for both raw RNA and DNA sequencing reads using *fastp* (v0.23.4) to remove adapters and low-quality sequences (Chen et al., 2018). For the RNA-seq data, *TFE3* fusion transcripts were identified using *STAR-Fusion* (v1.15.1) (Haas et al., 2017). For the DNA-seq data, paired-end reads were aligned to the human reference genome (GRCh37/hg19) using the *BWA-MEM* (v0.7.17). The resulting Sequence Alignment Map (SAM) files were converted to Binary Alignment Map (BAM) format, sorted, and indexed using *Samtools* (v1.17). Finally, the precise genomic breakpoints of the *TFE3* rearrangements were identified from the aligned DNA-seq data using *Factera* (v1.4.4) (Newman et al., 2014).

### 2.2 Breakpoint distribution and exon/intron length normalization

A chi-square (χ^2^) goodness-of-fit test was utilized to determine if the observed breakpoint distribution within the *TFE3* gene deviated significantly from a random distribution. The genomic coordinates, structure, and lengths of *TFE3* introns and exons were retrieved from GENCODE annotations corresponding to the human reference genome assembly GRCh37/hg19. Considering the oncogenic selective pressure of *TFE3* fusion proteins, it is sufficient to analyze whether the breakpoints in the *TFE3* gene are randomly distributed between exon 1 and intron 6. The null hypothesis (H_0_) stated that breakpoints were distributed randomly across upstream of exon 7 of *TFE3*, with the frequency in any given region being proportional to its length. The expected number of breakpoints (Eᵢ) for each region was calculated using the formula: Eᵢ = (Lᵢ/L_total) × N, where Lᵢ is the length of a given region, L_total is the cumulative length of upstream of exon 7 of *TFE3*, and N is the total number of observed breakpoints (N = 31). For the primary analysis, breakpoints were categorized into two groups for comparison: those within intron 5 and those located in all other genic regions (introns and exons) combined in upstream of exon 7 of *TFE3*. A *P*-value <0.05 was set as the threshold for statistical significance. Subsequently, to normalize for the varying lengths of these regions, breakpoint density was calculated for each feature as the number of breakpoints per kilobase (kb). This normalization enabled a direct comparison of breakpoint density across different genic regions.

### 2.3 Analysis of GC content

To investigate local GC content as a potential confounding factor in breakpoint localization, we calculated the GC percentage for all introns and exons of the *TFE3* gene. Genomic sequences for these regions were extracted from the human reference genome (GRCh37/hg19), and their respective GC contents were computed using the *nuc* utility within *bedtools*. This descriptive analysis aimed to determine if the breakpoint-dense intron 5 possessed anomalous GC content compared to other regions of the gene that are less prone to breakage.

### 2.4 Prediction of non-canonical DNA structures and computational pipeline for thermodynamic stability fluctuation analysis

To investigate the structural features of the *TFE3* gene, we performed two distinct computational analyses: a motif-based search for known non-canonical DNA structures and a comprehensive biophysical analysis of local thermodynamic stability.

#### 2.4.1 Motif-based prediction of G-Quadruplexes and palindromes

The genomic sequence for human *TFE3* (based on transcript NM_006521.6) was extracted from the human reference genome (GRCh37/hg19). We first scanned this sequence for motifs known to form non-canonical DNA structures that can impede replication fork progression. G-quadruplex Prediction: Putative G-quadruplex forming sequences were identified using two independent algorithms to ensure robustness. QGRS Mapper (parameters: max length 30, min G-group 2, loop size 0–36) was used for its established G-scoring algorithm. Predictions were cross-validated using G4Hunter. G-quadruplexes, were evident to be the reason behind TFE3 induced oncogenesis executed by translocation (Verma and Das, 2018), were predicted using multiple algorithms (Lerner and Sale, 2019). Both tools have been successfully applied in previous studies (Bezzi et al., 2021; Obara et al., 2024; Nie et al., 2023). Palindrome Prediction: Palindromic sequences, which can form cruciform or hairpin structures, were identified using the Palindrome analysis tool (parameters: min length 6 bp, max length 30 bp, spacer 0–10 bp, mismatches ≤1) and cross-validated with EMBOSS Palindrome (Lu et al., 2015).

#### 2.4.2 Computational pipeline for thermodynamic stability fluctuation analysis

To move beyond simple motif counts, we designed a computational pipeline to model the biophysical stability of the DNA sequence at high resolution. The entire pipeline, from sequence preparation to peak identification, is detailed below.

Step 1: Rationale and Sequence Preparation. Our central hypothesis is that transiently single-stranded DNA (ssDNA), which forms during replication and transcription, can fold into secondary structures. The stability of these structures can influence genomic integrity. To model this, we employed a sliding window approach across the *TFE3* gene sequence. A window size of 300 base pairs (bp) was selected to approximate the length of transiently exposed ssDNA, such as that seen in Okazaki fragments on the lagging strand (Hay and DePamphilis, 1982; Anderson and DePamphilis, 1979). The analysis advanced with a 1-nucleotide (nt) step size to generate a high-resolution, nucleotide-by-nucleotide profile of local folding potential.

Step 2: Calculation of Folding Free Energy (*ΔG*). For each 300-bp window, the minimum free energy of folding (*ΔG*, in kcal/mol) was calculated using the *Mfold* software (v3.6), which is widely used for predicting nucleic acid secondary structures. The *ΔG* value represents the thermodynamic stability of the most stable secondary structure a sequence can form; more negative values indicate more stable structures. Calculations were performed under simulated physiological conditions (temperature = 37 °C [Na+] = 1.0 M [Mg2+] = 0.0 M) (Zuker, 2003). Dillon et al. demonstrate the validity of *Mfold* secondary structure predictions (Dillon et al., 2013). The resulting ΔG for each window was assigned to the coordinate of its starting nucleotide, creating a comprehensive energy landscape across the gene. To ensure our findings were not tool-dependent, the entire analysis was independently validated using the *RNAfold* program (*ViennaRNA* package v2.4.7) with its corresponding DNA energy parameters.

Step 3: Quantification of Thermodynamic Stability Fluctuation (|d*ΔG*|). Breakpoints are hypothesized to occur not just in regions of high or low stability, but in regions where this stability changes sharply. To quantify this local structural volatility, we calculated the thermodynamic stability fluctuation, denoted as |d*ΔG*|. This metric was computed as the absolute difference in folding energy (ΔG) between two points separated by a 30-bp interval:

$$\left|d\Delta G_{i}\;\right|=\left|\Delta G_{i+30}-\Delta G_{i}\right|$$

where *ΔG*_*i*_ is the free energy of the 300-bp window starting at position *i*. A high |d*ΔG*| value thus signifies a rapid transition between regions of differing structural stability, which we propose as a marker for potential genomic fragility.

Step 4: Peak Identification and Association with Breakpoints. Significant peaks in the |d*ΔG*| profile, representing points of maximum structural stress, were identified objectively as extreme outliers using the robust interquartile range (IQR) method. A |dΔG| value was classified as a significant peak if it exceeded the upper threshold of Q3 + 3*IQR, where Q3 is the third quartile. Finally, a predicted thermodynamic stability fluctuation peak was considered to be associated with an experimental breakpoint if it was located within a ±500 bp window of the intron/exon where the breakpoint cluster is located. This window was chosen to account for the fact that the region of maximum structural stress may not be the precise site of breakage but rather predisposes the local chromatin environment to DNA damage. This comprehensive pipeline allowed us to correlate high levels of predicted structural stress with the locations of experimentally observed DNA breaks.

### 2.5 Statistical analysis

To rigorously assess the spatial association between breakpoint locations and specific genomic features, we employed a permutation testing framework. This non-parametric approach is particularly robust for datasets with a small number of regions and a highly skewed distribution of events (i.e., breakpoints), as it does not rely on assumptions of normality or other theoretical distributions.

The primary null hypothesis (H_0_) for our permutation tests was that the spatial distribution of the 31 observed breakpoints is independent of the underlying genomic feature being tested.

We performed separate permutation tests for three features: (1) the peak thermodynamic stability fluctuation (max |d*ΔG*|) within each genomic region (exons and introns upstream of exon 7), (2) G-quadruplex motifs density (motifs/kb) within each genomic region (exons and introns upstream of exon 7), and (3) palindromic motifs density (motifs/kb) within each genomic region (exons and introns upstream of exon 7).

For each feature, we defined a test statistic (T) that measures the degree of association between breakpoints and the feature’s value. This was calculated as the sum of the feature values across all regions, weighted by the number of breakpoints in each region:

$$T=\Sigma \;\left(Fᵢ\times Bᵢ\right)$$

where *Fᵢ* is the value of the feature (e.g., max |dΔG|) for region *i*, and *Bᵢ* is the number of observed breakpoints in region *i*. The observed test statistic, *T_obs*, was calculated using the actual experimental data. A high *T_obs* value suggests that breakpoints preferentially occur in regions with high feature values. To generate an empirical null distribution, we simulated 10,000 random permutations of the breakpoint locations. In each permutation, the 31 breakpoints were randomly redistributed among the 12 genomic regions (introns and exons from exon 1 to intron 6). Critically, to account for differences in region size, the probability of a breakpoint being assigned to a specific region *i* was proportional to its length (*Lᵢ*). This ensures that the null model represents a random distribution based on length alone. For each of the 10,000 permutations, a new test statistic, *T_perm*, was calculated using the randomly assigned breakpoint distribution. This process generated a null distribution of 10,000 *T_perm* values, representing the range of association strengths expected purely by chance. The one-sided P-value was calculated as the proportion of permutations where the permutation statistic was greater than or equal to the observed statistic:

$$P=\left(\text{Number }\text{of }\text{times }T\_\text{perm}\geq T\_\text{obs}\right)\;/\;10,000$$

A P-value <0.05 was considered statistically significant, leading to the rejection of the null hypothesis and indicating a significant spatial co-localization between breakpoints and the tested genomic feature. All permutation tests were implemented and performed using custom scripts in R (version 4.5.7).

### 2.6 Validation using public data (COSMIC)

Our analysis of the *TFE3* gene revealed a strong association between the breakpoint cluster in intron 5 and a region of high thermodynamic stability fluctuation, quantified as the rate of change in Gibbs free energy (|d*ΔG*|). To determine if this correlation represents a more general mechanism of translocation-mediated mutagenesis rather than a phenomenon unique to *TFE3*-rRCC, we sought to validate our hypothesis using an independent, large-scale dataset. We posited that if high |d*ΔG*| is a genuine marker of genomic fragility, then recurrent breakpoints in other frequently rearranged cancer-associated genes should also co-localize with peaks of thermodynamic stability fluctuation.

We queried the COSMIC Gene Fusion Curation resource (human somatic variants; COSMIC v102, released 21-MAY-25) and ranked genes by the number of samples in which fusion events were reported. Following the removal of duplicate entries from the same sample, we selected ten genes based on two criteria: harboring the highest number of total breakpoints and exhibiting a highly concentrated breakpoint distribution (defined as a primary hotspot containing >50% of the gene’s total breakpoints). The selected genes were *ABL1, ALK, CCDC6, ETV6, KIF5B, NCOA4, PBX1, RET, RUNX1*, and *TCF3*. In addition, due to the extremely high frequency of fusion events, we analyzed three pediatric sarcoma-associated genes: *EWSR1* (Ewing sarcoma), *NTRK3* (infantile fibrosarcoma, congenital mesoblastic nephroma), and *FOXO1* (alveolar rhabdomyosarcoma). Breakpoint coordinates were harmonized to a single reference (GRCh37/hg19). For each gene, we identified the genomic area with the highest frequency of breakpoints (defined as the primary breakpoint hotspot) and calculated its proportion relative to the total number of recorded breakpoints. Subsequently, the identical free energy analysis performed on *TFE3* (as described in Section 2.4) was applied to each gene. This entailed calculating local Gibbs free energy changes (*ΔG*) across their genomic sequences using *Mfold* software, deriving the corresponding |d*ΔG*| profile, and identifying significant peaks using the interquartile range (IQR) method. Finally, for each gene, we determined whether its primary breakpoint hotspot co-localized with a |d*ΔG*| peak by examining a 500 bp window centered on the hotspot. This window size, consistent with the methodology in Section 2.4, was selected to capture significant local thermodynamic stability fluctuation while accommodating gene-specific variations.

## 3 Results

### 3.1 Breakpoints of *TFE3* in *TFE3*-rRCC patients

Analysis of sequencing data from 31 patient cases revealed that RNA sequencing identified fusion breakpoints within specific introns for 25 cases (Table 1), whereas DNA sequencing pinpointed the precise fusion sites in the remaining six cases. Of these 31 fusion sites, 23 (74.19%) were located in intron 5, followed by four in intron 4, two in intron 1, one in exon 4, and one in exon 5. The high prevalence (74.19%) of breakpoints in intron 5 suggests that this region is a breakpoint hotspot.

**TABLE 1:** Fusion sites and putative breakpoint ranges of the fusion gene in *TFE3*-rRCC patients.

Patient	Fusion gene	DNA sequencing results of the *TFE3* ^a^		RNA sequencing results of the *TFE*3^a^		HGVS	Breakpoint location
		3′end fusion site	5′end fusion site	3′end fusion site	5′end fusion site		
1	*ASPSCR1::TFE3*	-	-	-	chrX:48895535	NM_006521.6:r.1017delins [*ASPSCR1*]	intro5
2	*ASPSCR1::TFE3*	-	chrX:48895908	chrX:48895639	-	NM_006521.6:r.912delins [*ASPSCR1*]	intro4
3	*ASPSCR1::TFE3*	chrX:48891945	chrX:48891946	-	chrX:48895535	NM_006521.6:r.1017delins [*ASPSCR1*]	intro5
4	*ASPSCR1::TFE3*	chrX:48895784	-	-	chrX:48895908	NM_006521.6:r.726delins [*ASPSCR1*]	exon4
5	*ASPSCR1::TFE3*	chrX:48892022	-	chrX:48891766	-	NM_006521.6:r.1017delins [*ASPSCR1*]	intro5
6	*ASPSCR1::TFE3*	-	-	chrX:48891766	chrX:48895535	NM_006521.6:r.1017delins [*ASPSCR1*]	intro5
7	*PRCC::TFE3*	chrX:48895666	chrX:48895668	-	-	NC_000023.11:g.48895667delins [*PRCC*]	intro4
8	*PRCC::TFE3*	-	-	chrX:48895639	-	NM_006521.6:r.912delins [*PRCC*]	intro4
9	*PRCC::TFE3*	-	-	chrX:48891766	chrX:48895535	NM_006521.6:r.1017delins [*PRCC*]	intro5
10	*PRCC::TFE3*	-	chrX:48895230	chrX:48891766	chrX:48895535	NM_006521.6:r.1017delins [*PRCC*]	intro5
11	*MED15::TFE3*	-	-	chrX:48891766	-	NM_006521.6:r.1017delins [*MED15*]	intro5
12	*MED15::TFE3*	-	chrX:48893163	chrX:48891766	-	NM_006521.6:r.1017delins [*MED15*]	intro5
13	*MED15::TFE3*	-	-	chrX:48891766	-	NM_006521.6:r.1017delins [*MED15*]	intro5
14	*MED15::TFE3*	chrX:48895306	-	-	-	NC_000023.11:g.48895307delins [*MED15*]	intro5
15	*SFPQ::TFE3*	-	-	chrX:48891766	-	NM_006521.6:r.1017delins [*SFPQ*]	intro5
16	*SFPQ::TFE3*	-	-	chrX:48898095	-	NM_006521.6:r.248delins [*SFPQ*]	intro1
17	*SFPQ::TFE3*	-	-	chrX:48895639	-	NM_006521.6:r.912delins [*SFPQ*]	intro4
18	*SFPQ::TFE3*	-	chrX:48891274	chrX:48891766	-	NM_006521.6:r.1017delins [*SFPQ*]	intro5
19	*SFPQ::TFE3*	chrX:48892310	chrX:48892305	-	-	NC_000023.11:g.48892311delins [*SFPQ*]	intro5
20	*SFPQ::TFE3*	chrX:48895555	chrX:48895568	-	-	NC_000023.11:g.48895556delins [*SFPQ*]	exon5
21	*SFPQ::TFE3*	chrX:48893347	chrX:48893356	chrX:48891766	-	NM_006521.6:r.1017delins [*SFPQ*]	intro5
22	*SFPQ::TFE3*	-	chrX:48895530	chrX:48891766	chrX:48895535	NM_006521.6:r.1017delins [*SFPQ*]	intro5
23	*EWSR1::TFE3*	-	-	chrX:48898095	-	NM_006521.6:r.248delins [*EWSR1*]	intro1
24	*NONO::TFE3*	chrX:48892028	-	chrX:48891766	chrX:48895535	NM_006521.6:r.1017delins [*NONO*]	intro5
25	*NONO::TFE3*	-	chrX:48895667	chrX:48891766	chrX:48895535	NM_006521.6:r.1017delins [*NONO*]	intro5
26	*NONO::TFE3*	chrX:48894609	-	-	-	NC_000023.11:g.48894610delins [*NONO*]	intro5
27	*NONO::TFE3*	-	chrX:48894282	chrX:48891766	chrX:48895535	NM_006521.6:r.1017delins [*NONO*]	intro5
28	*NONO::TFE3*	-	-	chrX:48891766	-	NM_006521.6:r.1017delins [*NONO*]	intro5
29	*NONO::TFE3*	-	chrX:48894455	-	-	[*NONO*] delinsNC_000023.11:g.48894455	intro5
30	*NONO::TFE3*	-	-	chrX:48891766	chrX:48895535	NM_006521.6:r.1017delins [*NONO*]	intro5
31	*NONO::TFE3*	-	-	chrX:48891766	chrX:48895535	NM_006521.6:r.1017delins [*NONO*]	intro5

^a^ The DNA-seq, data provides precise genomic coordinates; The RNA-seq, data identifies the involved exons/introns.

### 3.2 Breakpoint enrichment in intron 5 is independent of its length

Our null hypothesis posited that the observed distribution of breakpoints across intron 5 and all other genic regions upstream of exon 7 of *TFE3* is proportional to the length of these regions. The significance level was set at 0.05. The chi-square test revealed a highly significant deviation between the observed and expected breakpoint distributions (χ^2^ (1, N = 31) = 15.80, *P* < 0.001), leading us to reject the null hypothesis and concluding that intron 5 is a breakpoint hotspot region (Table 2).

**TABLE 2:** Breakpoints number of expected and observed of intron 5 with other introns/exons of *TFE3*.

*TFE3*	Expected breakpoint	Observed breakpoint
Intro5	12.19	23
Other introns and exons upstream of exon 7 of *TFE3*	18.81	8

To further characterize the breakpoint distribution, we calculated the standardized density (breakpoints per kilobase). Notably, this analysis revealed that intron 4 exhibited the highest breakpoint density (48.19/kb), followed by exon 5 (9.62/kb) and intron 5 (6.10/kb). Synthesizing these results, while intron 5 does not have the highest breakpoint *density*, it harbors the largest absolute number of breakpoints. Therefore, the critical finding is that intron 5 represents a significant hotspot of chromosomal fragility, accumulating a disproportionately high number of breakpoints relative to its size, even though other smaller regions exhibit higher breakpoint densities.

### 3.3 Breakpoint enrichment in intron 5 is not associated with its GC content

To evaluate GC content as a potential contributing factor to breakpoint distribution, we examined its percentage across all *TFE3* exons and introns. Our analysis revealed that breakpoint-rich intron 5 exhibited a GC content of 48.37%, a moderate value that lies within the range observed for other *TFE3* introns and exons (43.37%–70.73%; Table 3). No anomalous GC content was identified in intron 5 that would distinguish it from breakpoint-sparse regions of the gene. These findings suggest that local GC content is not a primary determinant of breakpoint susceptibility in *TFE3*.

**TABLE 3:** The AT/GC content percentage, A/C/G/T content, and fragment length for each intron and exon of the *TFE3* gene.

*TFE3*	AT percentage	GC percentage	Number of A	Number of C	Number of G	Number of T	Sequence length
exon1	0.292683	0.707317	39	105	69	33	246
intron1	0.479544	0.520456	674	744	579	545	2542
exon2	0.522124	0.477876	30	21	33	29	113
intron2	0.453677	0.546323	241	245	327	234	1,047
exon3	0.349835	0.650165	59	86	111	47	303
intron3	0.481203	0.518797	197	223	122	123	665
exon4	0.314286	0.685714	27	65	103	50	245
intron4	0.566265	0.433735	24	7	29	23	83
exon5	0.480769	0.519231	23	26	28	27	104
intron5	0.516317	0.483683	1,059	959	864	887	3,769
exon6	0.444444	0.555556	25	30	35	27	117
intron6	0.511364	0.488636	107	76	96	73	352
exon7	0.517857	0.482143	6	14	13	23	56
intron7	0.435484	0.564516	53	33	72	28	186
exon8	0.466667	0.533333	14	18	22	21	75
intron8	0.49089	0.50911	439	552	426	504	1921
exon9	0.394558	0.605442	20	45	44	38	147
intron9	0.5375	0.4625	231	180	190	199	800
exon10	0.427733	0.572267	390	514	559	412	1875

### 3.4 Distribution of non-canonical DNA structures and their stability

**FIGURE 2:**
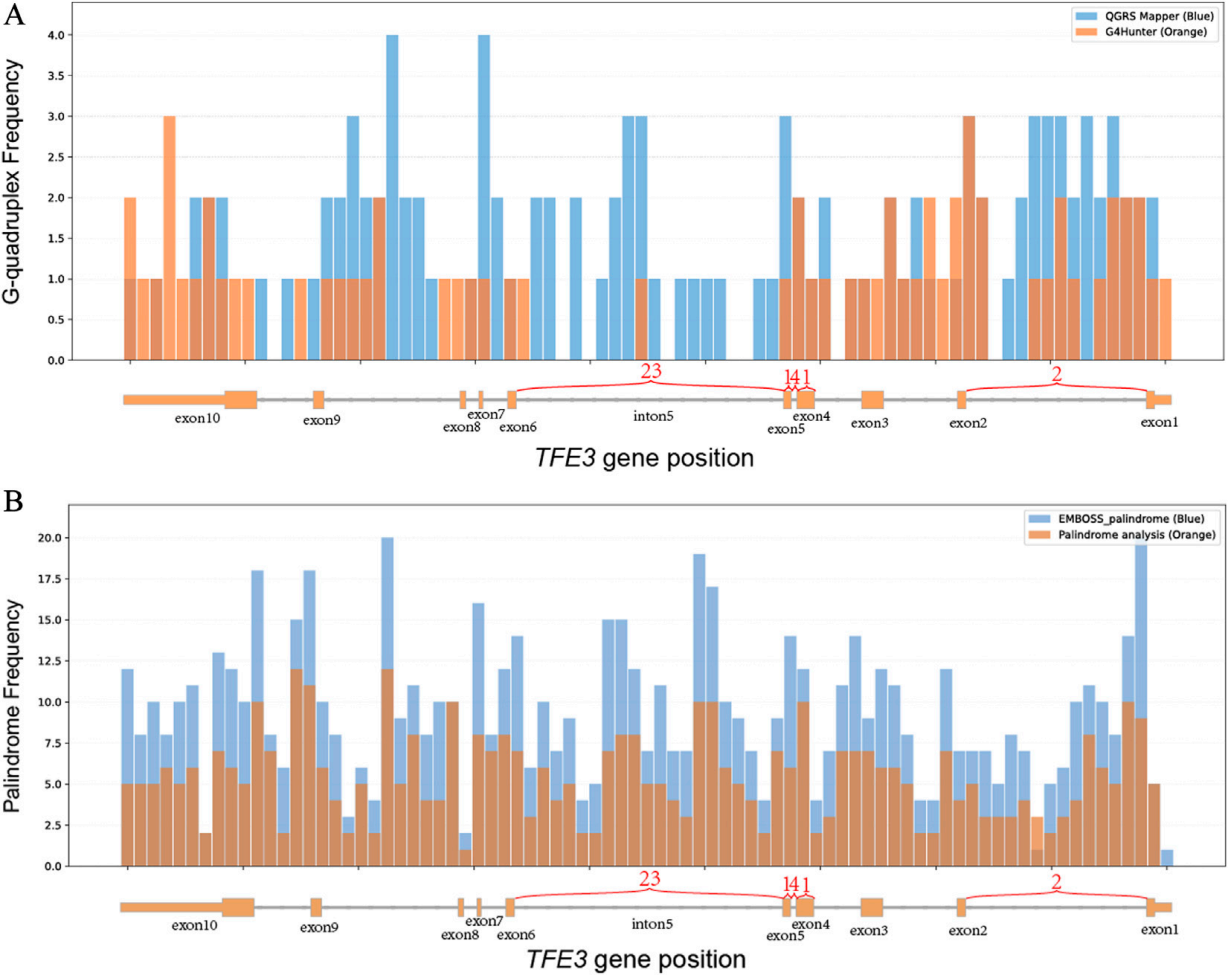
Histogram of the correspondence between the distribution of G-quadruple and palindrome counts on the *TFE3* gene and actual *TFE3* gene breakpoints. The X-axis represents the *TFE3* gene, showing the number of breakpoints across its exons and introns, the Y-axis represents the count (a: G-quadruple; b: palindrome), the histogram bin width is 184 bp; **(A)** light blue indicates the G-quadruple count distribution calculated using *QGRS Mapper*, and light orange indicates the G-quadruple count distribution calculated using G4Hunter. **(B)** light blue indicates the palindrome count distribution calculated using *Palindrome analysis*, and light orange indicates the palindrome count distribution calculated using *EMBOSS Palindrome*.

We predicted G-quadruples and palindromes within *TFE3*. Cross-validate by calculating G-quadruple sequences in *TFE3* with QGRS Mapper and G4Hunter, and by calculating palindromic structures in *TFE3* with Palindrome analysis and EMBOSS Palindrome. The number of DNA structures at various positions of the *TFE3* gene is shown in the figure (Figure 2).

**FIGURE 3:**
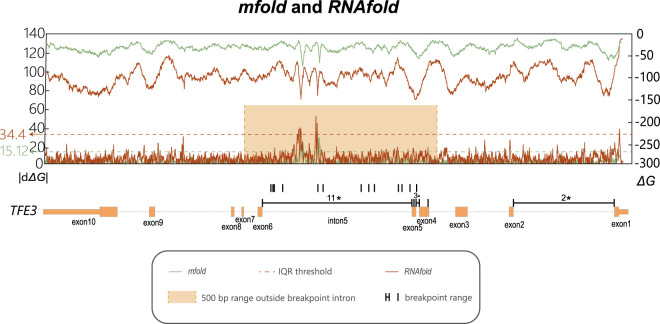
Line charts of *ΔG* and its rate of change within the *TFE3* gene. The x-axis indicates the genomic coordinates of the *TFE3* gene, along with the actual breakpoint location in each intron and exon, for or those without precise detection, the annotations indicate approximate locations. The orange box is the range after extending the regions with significant breakpoint enrichment outward by 500bp. The dashed line represents the threshold for peak detection calculated using the IQR method. Values above the blue line represent the |d*ΔG*| peaks, by defining values above Q3 + 3 * IQR as peaks. The upper line represents *ΔG* (secondary y-axis on the right), calculated using *Mfold* and *RNAfold* with a sliding window of 300bp and a step size of 1 bp. The lower line represents the rate of change in *ΔG*, calculated at various positions within the *TFE3* gene as the absolute difference in *ΔG* values across a fixed 30 bp window (|d*ΔG*|), shown on the primary y-axis on the left. The green line: calculated by *Mfold*; The red line calculated by *RNAfold*.

To quantify the thermodynamic stability of potential secondary structures, the free energy of folding (*ΔG*) was calculated using *Mfold*. This was performed on the full-length sequence of *TFE3* using a 300-nucleotide (nt) sliding window with a 1-nt step size, with the results presented in Figure 3 green line. To assess the thermodynamic stability fluctuation, we calculated the absolute change in *ΔG* over a 30-base-pair sliding window (|d*ΔG*|). The primary |d*ΔG*| peak was located precisely within intron 5, indicating a region of rapid change in thermodynamic stability. This pronounced thermodynamic stability fluctuation is hypothesized to contribute to the elevated frequency of DNA breakage at this locus.

To validate that the pronounced ΔG fluctuation in intron 5 was not an algorithmic artifact, the analysis was replicated using the *RNAfold* tool. These analyses corroborated our initial findings (Figure 3 orange line). Using the IQR method to identify peaks, defining extended 500 bp outward of intron 5 near the breakpoint. Although the absolute ΔG values varied slightly between the methods, all computational approaches consistently identified intron 5 as the region with the maximal |dΔG| peak.

### 3.5 Permutation testing reveals a specific association between breakpoint locations and thermodynamic instability fluctuation

All *TFE3* fusion genes retain exons 7 to 10; therefore, our analysis focuses only on the genes upstream of exon 7. To formally test the hypothesis that breakpoint hotspots are associated with specific genomic features, we used a permutation-based statistical framework. This approach allowed us to assess whether the observed co-localization of breakpoints with high |dΔG| peaks, or with G-quadruplex and palindromic motifs, was statistically significant or merely due to chance.

**FIGURE 4:**
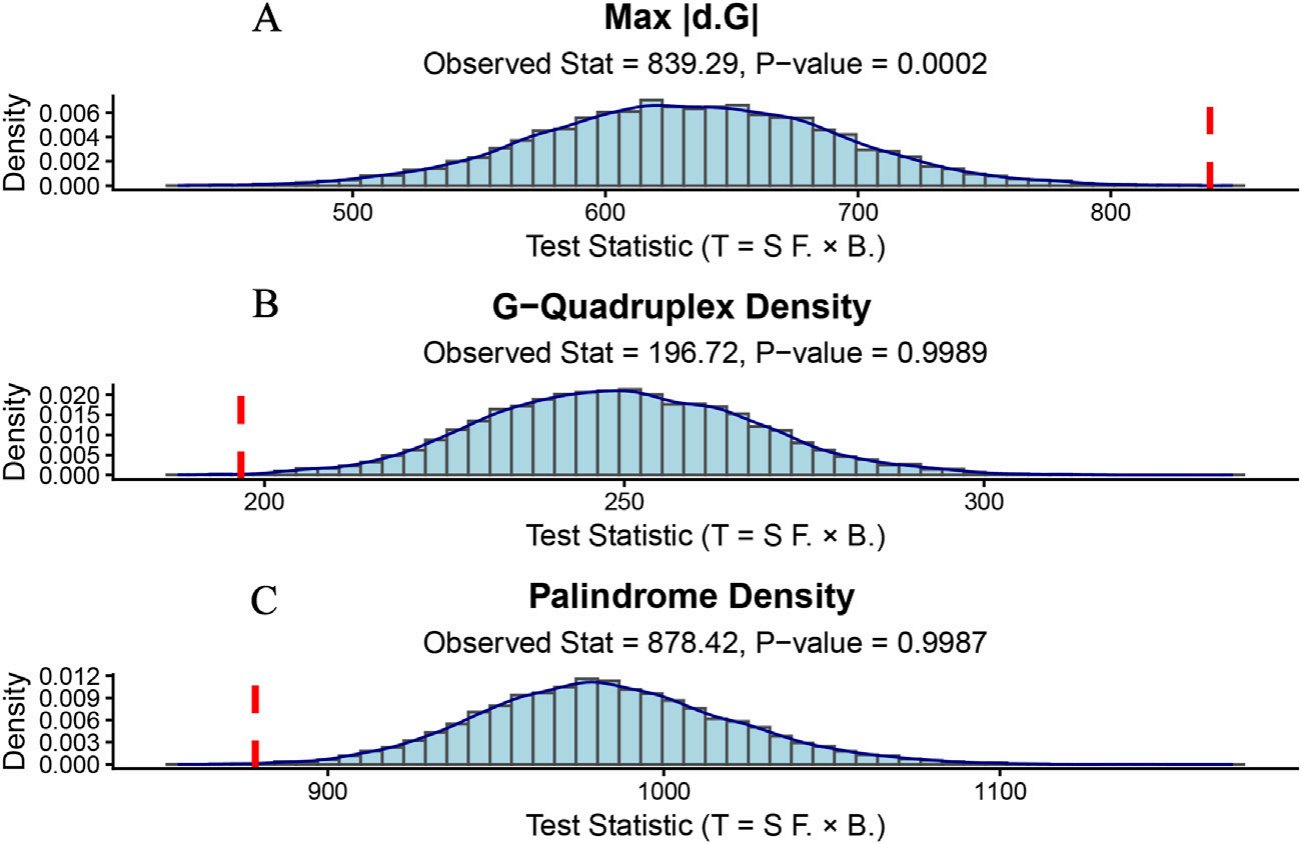
Permutation test for the association between breakpoint locations and genomic features. Histograms show the empirical null distribution of the test statistic (*T_perm*) from 10,000 permutations for **(A)** max |dΔG|, **(B)** G-quadruplex density, and **(C)** palindrome density. The observed test statistic (*T_obs*) is indicated by a red vertical line. **(A)** The observed association between breakpoints and max |dΔG| is significantly greater than expected by chance (P = 0.0002). **(B,C)** The observed associations for G-quadruplex and palindrome counts are not statistically significant (P = 0.9989 and P = 0.9987, respectively), indicating the correlation is weaker than expected under a random distribution.

The permutation test for the peak thermodynamic stability fluctuation (max |dΔG|) yielded a highly significant result. The observed test statistic (*T_obs*), which quantifies the weighted sum of |dΔG| values at breakpoint locations, was found to be in the extreme tail of the empirical null distribution generated from 10,000 permutations (P = 0.0002; Figure 4A). This provides robust evidence that breakpoints in *TFE3* are non-randomly drawn to areas with the greatest local changes in DNA secondary structure stability.

In contrast, when we applied the same permutation test to the quantity of non-canonical DNA structure motifs, we found no evidence of a positive association. The analyses for G-quadruplex density (*P* = 0.9989; Figure 4B) and palindromic structure density (*P* = 0.9987; Figure 4C) yielded P-values approaching 1.0, indicating that the observed association between breakpoints and these motif counts was even weaker than what would be expected under a random distribution (Table 4).

**TABLE 4:** Table of G-quadruple density, palindrome density, and maximum rate of change in *ΔG* for each intron and exon of *TFE3*.

Region	Length (bp)	Breakpoint count	Max |d*ΔG*| (kcal/mol)	G-quadruplexs count	Palindrome count	G-quadruplexs density (per kb)	Palindrome density (per kb)
exon1	246	0	10.83	2	10	8.13	40.65
intro1	2542	2	15.67	29	85	11.41	33.44
exon2	113	0	10.65	2	6	17.70	53.10
intro2	1,047	0	11.39	6	25	5.73	23.88
exon3	303	0	7.94	1	12	3.30	39.60
intro3	665	0	8.97	4	18	6.02	27.07
exon4	245	1	11.41	2	14	8.16	57.14
intro4	83	4	7.08	0	1	0.00	12.05
exon5	104	1	9.22	2	3	19.23	28.85
intro5	3,769	23	33	24	111	6.37	29.45
exon6	117	0	5.91	0	5	0.00	42.74
intro6	352	0	6.33	5	14	14.20	39.77
exon7	56	0	3.65	1	3	17.86	53.57
intro7	186	0	7.35	2	3	10.75	16.13
exon8	75	0	4.61	0	3	0.00	40.00
intro8	1921	0	9.31	19	56	9.89	29.15
exon9	147	0	7.3	2	3	13.61	20.41
intro9	800	0	7.85	1	15	1.25	18.75
exon10	1875	0	10.47	9	55	4.80	29.33

Taken together, these rigorous statistical tests confirm our primary hypothesis: the breakpoint hotspot in *TFE3* intron 5 is significantly associated with a biophysical property—high thermodynamic stability fluctuation—rather than a simple enrichment of G-quadruplex or palindromic sequence motifs.

This finding prompted us to validate this principle in a larger set of cancer-associated genes from the COSMIC database.

### 3.6 Validation using the COSMIC database

**FIGURE 5:**
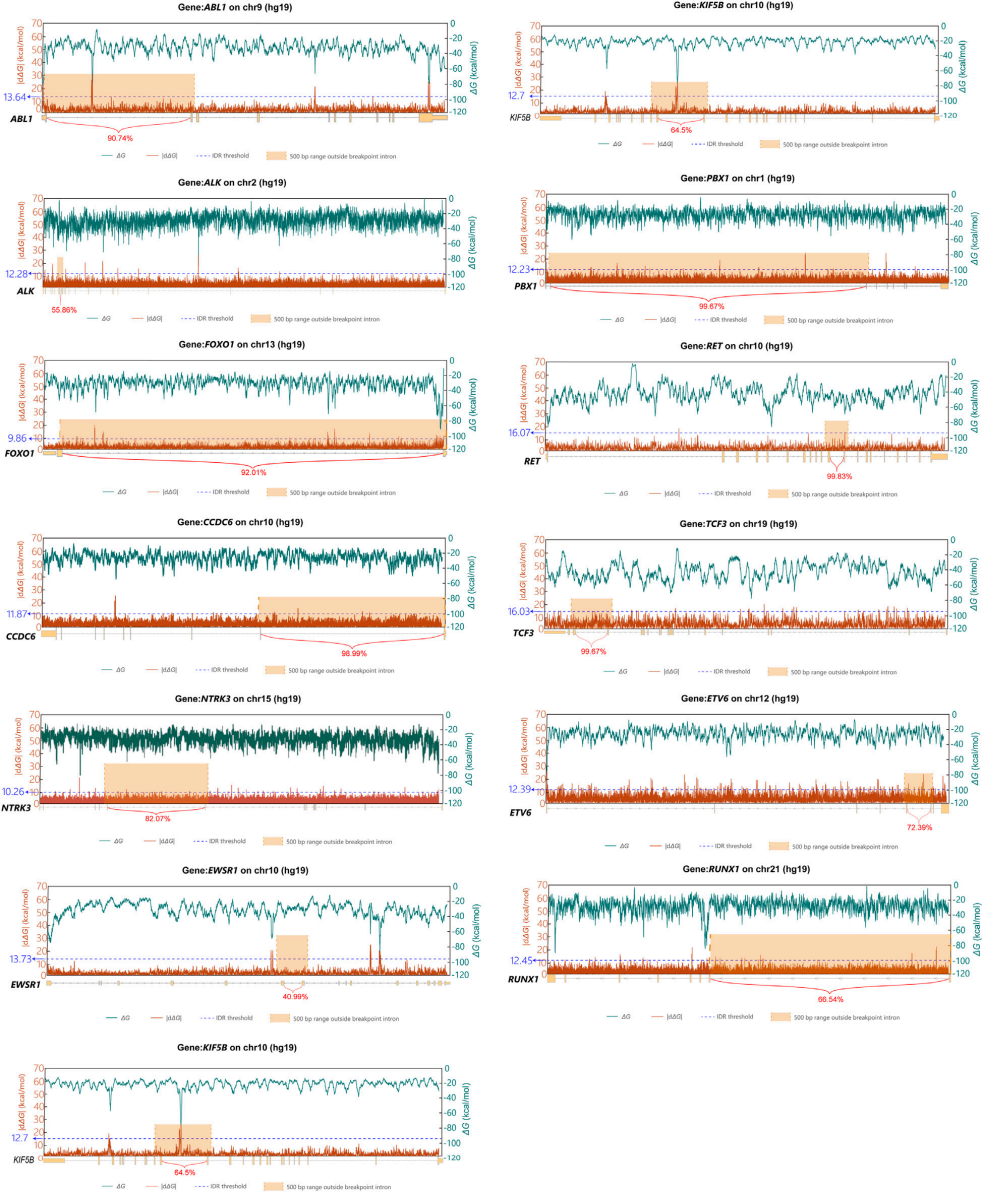
Line charts of *ΔG* and its rate of change within the twlve genes in the COSMIC database. The x-axis indicates the genomic coordinates of the twlve genes, along with introns enriched with breakpoints and the percentage of their breakpoint sites. The orange box is the range after extending the regions with significant breakpoint enrichment outward by 500bp. Values above the blue line represent the |d*ΔG*| peaks, by defining values above Q3 + 3 * IQR as peaks. The green line represents *ΔG*, shown on the secondary y-axis on the right, calculated using *Mfold* with a sliding window of 300bp and a step size of 1 bp. The red line represents the rate of change in *ΔG*, calculated at various positions within the twlve genes as the absolute difference in *ΔG* values across a fixed 30 bp window (|d*ΔG*|), shown on the primary y-axis on the left.

To validate these instability profiles, we correlated them with COSMIC-annotated breakpoint locations (Figure 5). This analysis revealed that for twelve of the thirteen genes investigated (*ABL1, ALK, CCDC6, ETV6, KIF5B, NCOA4, PBX1, RET, RUNX1, TCF3, FOXO1*, and *NTRK3*), prominent |d*ΔG*| peaks coincided with regions enriched with breakpoints. In contrast, *EWSR1*, the sole exception, did not exhibit a distinct |d*ΔG*| peak within its breakpoint-enriched region. This latter observation is consistent with the understanding that breakpoint formation is a multifactorial process, influenced by various factors beyond thermodynamic stability fluctuation.

## 4 Discussion

Genomic instability, characterized by an increased rate of mutations and chromosomal rearrangements, is a hallmark of many pathological disorders. However, it also plays a vital role in driving evolution. The propensity for such rearrangements is influenced by two opposing sets of factors. The first category comprises factors that promote genomic stability, such as the replication machinery, DNA repair systems, and S-phase checkpoint regulators. The second comprises factors that predispose the genome to instability, including endogenous sources such as fragile sites and regions of highly transcribed DNA sequences.

In *TFE3*-rearranged renal cell carcinoma (*TFE3*-rRCC), chromosomal breakage occurs consistently at the *TFE3* gene locus. Fragile sites are inherently vulnerable to chromosomal breakage, and previous studies have mapped *TFE3* to the fragile site FRAXG. To more precisely map the breakpoints, we sequenced the *TFE3* gene in a cohort of 31 patients diagnosed with *TFE3*-rRCC. Our analysis revealed that in 23 patients (74.19%), the breakpoints clustered within intron 5 of *TFE3*. This finding establishes that *TFE3* intron 5 is a specific breakpoint hotspot in *TFE3*-rRCC.

Crucially, this observed clustering is not a statistical artifact. A chi-square analysis statistically confirmed that the predisposition of intron 5 to breakage is an intrinsic feature of this region, rather than a consequence of its large genomic size. This robust statistical evidence allowed us to reject the null hypothesis of random breakage, implicating specific underlying biological mechanisms. Furthermore, we explored other potential sequence-based factors, such as GC content, which has been previously linked to genomic instability. Our analysis revealed that the GC content of intron 5 was moderate and unexceptional compared to other introns within the *TFE3* gene. This finding suggests that simple base composition is unlikely to be the primary driver of the observed fragility, prompting a search for more complex structural determinants.

Aphidicolin (APH)-induced common fragile sites are known to contain a higher density of sequences with the potential to form secondary structures compared to non-fragile regions of the genome (Dillon et al., 2013). To investigate this possibility, we first predicted the distribution of G-quadruplex and palindromic motifs. To formally test the association, we employed a rigorous permutation test. This analysis confirmed the lack of a statistically significant positive association between breakpoint locations and the quantity of either G-quadruplexes (*P* = 0.9989) or palindromic sequences (*P* = 0.9987). This lack of a strong correlation does not definitively rule out their involvement. The algorithmic prediction of these structures is inherently dependent on specific sequence patterns and scoring thresholds. Therefore, it is plausible that non-canonical or transient structures, not detected by these algorithms, still contribute to instability in this region (Verma and Das, 2018).

This led us to hypothesize that biophysical properties, rather than simple sequence motifs, could be the driving factor. We modeled DNA secondary structure thermodynamic stability across the gene and quantified its local fluctuation as |d*ΔG*|. This analysis revealed a dramatic |d*ΔG*| peak precisely within the intron 5 breakpoint hotspot (Figure 3). To validate whether this striking visual co-localization was statistically meaningful, we utilized our permutation testing framework. The test revealed a highly significant spatial correlation between breakpoint locations and the peaks of thermodynamic stability fluctuation (*P* = 0.0002). This robust statistical validation supports our central conclusion: the fragility of intron 5 is intrinsically linked to its landscape of thermodynamic stability flutuation, where rapid transitions between structurally stable and unstable states occur.

Using the same approach to analyze oncogenic fusion genes from the COSMIC somatic gene rearrangement database, we found that the consistent co-localization of breakpoints with regions of high thermodynamic stability fluctuation across a diverse set of well-established cancer genes provides powerful, independent support for our central hypothesis. This analysis demonstrates that the principle linking a high thermodynamic stability fluctuation to genomic fragility is not an isolated finding specific to *TFE3*. Instead, it appears to be a broader phenomenon contributing to non-random breakpoint formation in oncogenesis. This finding serves as a biological validation of the thermodynamic model developed previously, suggesting that fluctuations in the energy landscape of DNA secondary structures are a key influencing factor of genomic instability and a predisposing factor for chromosomal translocations.

The primary strengths of this study are threefold. First, we identified that *TFE3* breakpoints in *TFE3*-rRCC are predominantly concentrated in intron 5, pinpointing a specific hotspot for this rearrangement. Second, whereas previous thermodynamic analyses of fragile sites were typically conducted at the gene level or larger genomic scales, our study provides a high-resolution analysis at the intron/exon level. Third, our findings provide strong support for an energy-based mechanism, linked to thermodynamic stability fluctuation in DNA secondary structure, as a plausible biophysical feature contributing to the propensity for breakage at this fragile site.

This study also has several limitations. First, our reliance on bioinformatics tools means our findings are inherently predictive. Consequently, the identification of G-quadruplexes, palindromic sequences, and stable secondary structures is subject to potential false positives and negatives, and experimental validation remains technically challenging. Second, the use of data from the COSMIC database, which aggregates studies with variable breakpoint resolution and sampling depth, may introduce ascertainment bias. Third, DNA fragility is a multifactorial process influenced by mechanisms beyond stable non-B DNA structures, including replication origin density, R-loop formation, replication timing, and S-phase checkpoint regulation. Our study did not explore the interplay between these factors and our thermodynamic model. Future work should aim to integrate our model with experimental data from DRIP-seq, repli-seq, and other relevant assays to enable a more comprehensive investigation. Moreover, our findings remain correlative, and the precise mechanisms by which *ΔG* fluctuations might lead to DNA breakage require experimental validation. Future studies using functional genomics assays, such as for revealing DNA secondary structures (e.g., S1-END-seq) or R-loops (e.g., DRIP-seq) in cells under replicative stress, are needed to confirm the formation of these structures *in vivo* and causally link them to the breakage events that initiate oncogenic translocations (Matos-Rodrigues et al., 2022; Sanz and Chédin, 2019).

## Data Availability

The original contributions presented in the study are included in the article/Supplementary Material, further inquiries can be directed to the corresponding author.

## Abbreviations

*TFE3*, Transcription Factor Binding to IGHM Enhancer 3; *TFE3*-rRCC, Transcription Factor Binding to IGHM Enhancer 3 rearranged renal cell carcinoma; *ΔG*, Gibbs free energy change; IQR, Interquartile Range.
