# Supplementary material for: Mechanistic study of TFE3 breakage in TFE3-rearranged renal cell carcinoma: the perspective of non-canonical DNA structures and their stability
Source: Front Genet. 2025 Nov 25;16:1694739. doi: 10.3389/fgene.2025.1694739 (PMC12685264; doi:10.3389/fgene.2025.1694739)
Supplement: Supplementary file 1 [file Table1.docx]

Supplementary file 1: Chromosomal Fragile Sites of *TFE3* and Its Partner Genes in *TFE3*-rRCC

| number | broken gene | HGVS | fragile sites involved | chromosomal location |
| --- | --- | --- | --- | --- |
| 1 | *TFE3* | - | FRAXG | Xp11.2 |
| 2 | *ASPL(ASPSCR1 )* | *TFE3::ASPL(ASPSCR1)* | FRA17E | 17q24-25 |
| 3 | *PRCC* | *TFE3::PRCC* | FRA1F | 1921 |
| 4 | *SFPQ(PSF)* | *TFE3::SFPQ(PSF)* | FRA1 | 1p34 |
| 5 | *CLTC* | *TFE3::CLTC* | FRA17B | 17q23.1 |
| 6 | *PARP14* | *TFE3::PARP14* | FRA3M | 3q21 |
| 7 | *LUC7L3* | *TFE3::LUC7L3* | FRA17D | 17q21 |
| 8 | *KHSRP* | *TFE3::KHSRP* | FRA19A | 19q13 |
| 9 | *DVL2* | *TFE3::DVL2* |  |  |
| 10 | *MED15* | *TFE3::MED15* |  |  |
| 11 | *ARID1B* | *TFE3::ARID1B* | FRA6M | 6q25 |
| 12 | *MATR3* | *TFE3::MATR3* |  |  |
| 13 | *FUBP1* | *TFE3::FUBP1* | FRA1L | 1p31 |
| 14 | *NEAT1* | *TFE3::NEAT1* |  |  |
| 15 | *KAT6A* | *TFE3::KAT6A* |  |  |
| 16 | *EWSR1* | *TFE3::EWSR1* | FRA22B | 22q12.2 |
| 17 | *SETD1B* | *TFE3::SETD1B* | FRA12E | 12q24 |
| 18 | *ZC3H4* | *TFE3::ZC3H4* | FRA19A | 19q13 |
| 19 | *NONO(p54nrb)* | *TFE3::NONO(p54nrb)* |  |  |
| 20 | *RBM10* | *TFE3::RBM10* | FRAXH | Xp11/q11 |
| 21 | *GRIPAP1* | *TFE3::GRIPAP1* | FRAXH | Xp11/q11/ |
| 22 | *PTPN12* | *TFE3::PTPN12* | FRA7J | 7q11.23 |
| 23 | *ZNF627* | *TFE3::ZNF627* |  |  |
| 24 | *U2AF2* | *TFE3::U2AF2* | FRA19A | 19q13 |
| 25 | *SRRM2* | *TFE3::SRRM2* |  |  |
